# Association between advanced lung cancer inflammation index and distant metastasis in breast cancer: a retrospective cohort study

**DOI:** 10.3389/fonc.2025.1613346

**Published:** 2025-08-19

**Authors:** Xiao-dong Luan, Qian Ren, Ya-lin Zhang, Wen-hai Zhang, Chun-feng Liang, Si-zhi Liang, Zi-hao Liu, Yu-kun Liu

**Affiliations:** ^1^ Department of Breast Surgery, Qingdao Central Hospital, University of Health and Rehabilitation Sciences, Qingdao, Shandon, China; ^2^ Department of Breast Surgery, Guangxi Medical University Cancer Hospital, Nanning, Guangxi, China; ^3^ Department of Gastrointestinal Surgery, Guangxi Medical University Cancer Hospital, Nanning, Guangxi, China

**Keywords:** breast cancer, advanced lung cancer inflammation index (ALI), Distant metastasis (DM), prognostic biomarker, Retrospective cohort study

## Abstract

**Objective:**

This study aims to assess the relationship between the Advanced Lung Cancer Inflammation Index (ALI) and the risk of distant metastasis in breast cancer. While ALI is commonly used to evaluate the prognosis of lung cancer patients, its application in breast cancer and its correlation with distant metastasis are not well understood. Therefore, this study explores the potential of ALI as a predictor of distant metastasis in breast cancer patients.

**Methods:**

This retrospective study included 348 breast cancer patients, of whom 163 had distant metastasis. Patient demographic information, tumor characteristics, and ALI values were collected. Multivariate regression analysis was used to adjust for confounding factors, and dose-response analysis was performed to investigate the relationship between ALI and the risk of distant metastasis. The optimal ALI cutoff value was determined, and its predictive performance was evaluated.

**Results:**

The analysis showed that patients with lower ALI had a significantly higher risk of distant metastasis. Adjusted multivariate analysis revealed that for every one interquartile range (IQR) increase in ALI, the risk of distant metastasis in breast cancer decreased by 31% (OR=0.69, 95% CI 0.58-0.81). Dose-response analysis indicated a linear relationship between ALI and metastasis risk. The optimal ALI cutoff value was identified as 36.39 using the Youden index, with an area under the ROC curve (AUC) of 0.605, indicating moderate predictive power of ALI for distant metastasis in breast cancer.

**Conclusion:**

Lower ALI is significantly associated with an increased risk of distant metastasis in breast cancer. ALI may serve as a valuable predictor of distant metastasis, offering clinicians a new tool to better identify high-risk patients and facilitate early intervention. However, further prospective studies are required to validate its clinical utility.

## Background

Breast cancer is one of the most common malignancies affecting women worldwide, significantly impacting women’s health. As of 2022, breast cancer ranks second in incidence and fourth in mortality among cancers globally (1). In China alone, there are over 410,000 new breast cancer cases and more than 110,000 related deaths each year (2). According to data from the Surveillance, Epidemiology, and End Results (SEER) program, the 5-year survival rate for stage IV breast cancer in the U.S. is approximately 32% (3). In developing and underdeveloped countries, survival rates for metastatic breast cancer are even lower. In sub-Saharan Africa, according to the most recent multi-country population-based registry study in 2021, 20.8% of breast cancer patients had distant metastasis at diagnosis, approximately 46.4% of patients were diagnosed with locally advanced or metastatic disease, and the 5-year survival rate for metastatic breast cancer patients was less than 25% (4). Gagate et al. predict that by 2030, there will be an estimated 246,194 cases of metastatic breast cancer in the U.S., a 54.8% increase from the 158,997 cases estimated in 2015 (5). This rising trend underscores the importance of addressing metastatic breast cancer.

Most studies agree that metastatic breast cancer leads to poorer prognoses and higher medical costs, though diagnostic rates for metastatic breast cancer vary by region. In the U.S., stage IV breast cancer accounts for 6-10% of all breast cancer diagnoses, while in developing and underdeveloped regions, deaths from stage IV breast cancer pose an even more severe public health threat. In developed countries, delayed diagnoses mainly affect younger women, who may be at lower suspicion for cancer, and rural women, who may have less access to healthcare. In underdeveloped countries, however, delayed diagnoses affect women of all ages (6).

Currently, the gold standard for diagnosing breast cancer with distant metastasis is pathological examination. However, in practice, imaging techniques are often used to diagnose distant metastasis in breast cancer, including planar bone scintigraphy (BS), SPECT bone scans, CT, and conventional MRI. The use of hybrid imaging techniques, such as PET-CT and PET-MRI, is also increasing (7). Compared to imaging, laboratory tests offer the advantages of lower cost, greater accessibility, and the absence of radiation exposure. However, there is no standardized laboratory test for metastatic breast cancer. Traditional lab tests include tumor markers such as carcinoembryonic antigen (CEA), CA125 (8), and CA153 (9), and elevated blood calcium and alkaline phosphatase levels may also indicate distant metastasis (10). Another study showed that serum CK-MB (creatine kinase-MB, traditionally used as a cardiac biomarker) activity increases in multiple cancers, including breast cancer, with significantly higher serum CK-MB levels in patients with metastatic tumors compared to those with primary tumors (11). While CK-MB is not routinely used in clinical practice for breast cancer metastasis detection, this finding exemplifies how various laboratory parameters beyond conventional tumor markers might provide additional diagnostic insights.

These markers suggest that laboratory indicators may be able to more accurately identify breast cancer patients at risk for distant metastasis without relying on expensive imaging tests. This could help address disparities in healthcare access and enable more personalized treatment.

The Advanced Lung Cancer Inflammation Index (ALI), calculated as Body Mass Index (kg/m²) × Serum Albumin (g/dL)/Neutrophil-to-Lymphocyte Ratio, has been identified as a strong prognostic marker for overall survival in lung cancer patients (12). Despite its name suggesting lung cancer specificity, ALI integrates nutritional status (BMI and albumin) and systemic inflammation (neutrophil-to-lymphocyte ratio), making it potentially applicable across various cancer types. In a single-center study, Jafri et al. found that an ALI <18 at diagnosis was an independent predictor of poor outcomes in patients with advanced non-small cell lung cancer (NSCLC) (13). He et al. studied the relationship between ALI and small cell lung cancer prognosis, concluding that lower ALI was significantly associated with worse overall survival (OS) in these patients. Assessing ALI can help identify patients with poor prognosis, making it a useful biomarker in clinical practice (14). Similar results have been found in other cancer types. In a meta-analysis, Hua et al. demonstrated a statistically significant association between low ALI and worse OS across various cancer types (HR = 1.70, 95% CI = 1.41-1.99, P < 0.001). Subgroup analyses showed that ALI had significant prognostic value in non-small cell lung cancer, small cell lung cancer, colorectal cancer, head and neck squamous cell carcinoma, and diffuse large B-cell lymphoma (15).

However, the direct association between ALI and breast cancer remains unclear, particularly in terms of its diagnostic significance. This study, based on real-world data, examines ALI as both a continuous and categorical variable, uses multivariate analysis to control for confounding factors, and performs stratified comparisons. The objective is to evaluate the potential of ALI in predicting the risk of distant metastasis in breast cancer patients.

Ultimately, our goal is to stratify the risk of distant metastasis in breast cancer patients. This would assist clinicians, especially breast cancer specialists in primary care, in making informed decisions, reducing unnecessary medical interventions, and significantly improving patients’ quality of life.

## Materials and methods

### Participants

This retrospective study utilized data that was approved by the institutional review boards at our center. All methods were performed in accordance with the relevant guidelines and regulations. The inclusion criteria were: (1) a confirmed diagnosis of primary breast cancer, with or without distant metastasis beyond axillary lymph nodes; (2) completion of clinical blood marker tests prior to treatment (radiotherapy, chemotherapy) or surgical resection; (3) normal growth and development, with no history of rickets, acromegaly/gigantism, or acute/chronic kidney disease or inflammation; (4) no history of abnormal liver function or blood test results; (5) no other primary cancers. Exclusion criteria included: (1) distant metastasis occurring after treatment (surgical resection or chemotherapy); (2) lack of liver function or blood test results in clinical blood markers; (3) age under 18 years; (4) inability to obtain height and weight measurements due to bedridden conditions or other reasons; (5) presence of distant metastasis that could not be clinically confirmed; (6) patients with active autoimmune diseases (including but not limited to rheumatoid arthritis, systemic lupus erythematosus, inflammatory bowel disease, or other conditions requiring immunosuppressive therapy) that could affect inflammatory markers. The study included 348 breast cancer cases, with 185 diagnosed as non-metastatic and 163 as metastatic. Baseline characteristics of the study population are detailed in **Table 1**.

**Table 1:** Characteristics of the participants at baseline.

Characteristic	Group		*P* value
	Non-distant metastasis (N = 185)	Distant metastasis (N = 163)	
Age (years),Mean ± SD	47 ± 9	51 ± 11	0.003
Education, n (%)			0.002
Primary School	22 (11.9%)	33 (20.2%)	
Middle School	36 (19.5%)	43 (26.4%)	
High School	23 (12.4%)	13 (8.0%)	
Undergraduate	53 (28.6%)	22 (13.5%)	
Others	51 (27.6%)	52 (31.9%)	
Insurance, n (%)			0.007
No	34 (18.4%)	50 (30.7%)	
Yes	151 (81.6%)	113 (69.3%)	
Hypertension, n (%)			0.006
No	171 (92.4%)	135 (82.8%)	
Yes	14 (7.6%)	28 (17.2%)	
Diabetes, n (%)			0.671
No	174 (94.1%)	155 (95.1%)	
Yes	11 (5.9%)	8 (4.9%)	
Marital_status, n (%)			0.093
Married	171 (92.4%)	149 (91.4%)	
Unmarried	12 (6.5%)	6 (3.7%)	
Widow	1 (0.5%)	6 (3.7%)	
Divorced	1 (0.5%)	2 (1.2%)	
Location, n (%)			0.002
Village	69 (37.3%)	88 (54.0%)	
City	116 (62.7%)	75 (46.0%)	
Menstrual status, n (%)			0.003
Postmenopausal	73 (39.5%)	90 (55.2%)	
Premenopausal	112 (60.5%)	73 (44.8%)	
Pathological type, n (%)			0.695
Invasive ductal carcinoma	175 (94.6%)	152 (93.3%)	
Invasive lobular carcinoma	4 (2.2%)	6 (3.7%)	
Others	6 (3.2%)	5 (3.1%)	
Histological grade, n (%)			0.973
2 (Moderately differentiated)	145 (78.4%)	128 (78.5%)	
3 (Poorly differentiated)	40 (21.6%)	35 (21.5%)	
Subtype, n (%)			<0.001
HR+/HER2-	56 (30.3%)	89 (54.6%)	
HR+/HER2+	57 (30.8%)	35 (21.5%)	
HR-/HER2-	39 (21.1%)	10 (6.1%)	
HR-/HER2+	33 (17.8%)	29 (17.8%)	
Albumin (g/dl), Mean ± SD	4.07 ± 0.54	3.88 ± 0.44	<0.001
Neutrophil (10^9/l), Mean ± SD	4.03 ± 1.55	4.53 ± 2.80	0.046
Lymphocyte (10^9/l), Mean ± SD	1.88 ± 0.58	1.84 ± 0.73	0.605
BMI, Mean ± SD	23.8 ± 3.2	22.9 ± 3.3	0.018
ALI, Mean ± SD	50 ± 25	43 ± 25	0.005
Quartiles of ALI, n (%)			0.002
Q1	31 (16.8%)	56 (34.4%)	
Q2	51 (27.6%)	36 (22.1%)	
Q3	49 (26.5%)	38 (23.3%)	
Q4	54 (29.2%)	33 (20.2%)	

ALI, advanced lung cancer inflammation index; BMI, body mass index.

All patients underwent pathological examinations before treatment, including biopsy and partial resection biopsy, confirming invasive breast cancer. Distant metastasis was confirmed through whole-body computed tomography, magnetic resonance imaging, positron emission tomography-computed tomography, or pathological biopsy. Data on demographics, basic clinical and pathological information, body measurements, complete blood count, and serum albumin levels at diagnosis were extracted from patient medical records. ALI was calculated using the formula: ALI = Body Mass Index (kg/m²) * Serum Albumin (g/dL)/Neutrophil-to-Lymphocyte Ratio (16).

### Laboratory analysis

#### Blood sample collection

All venous blood samples were collected in the early morning after an overnight fast of at least 8 hours by trained nurses following standardized phlebotomy procedures to minimize pre-analytical variability. Two types of blood samples were collected from each participant: EDTA anticoagulated whole blood for complete blood count analysis and serum samples for albumin measurement.

#### Laboratory methods

Complete blood count, including neutrophil and lymphocyte counts, was performed using the Mindray CAL8000plus automated hematology pipeline system (Mindray, Shenzhen, China). All samples were processed within 2 hours of collection to ensure sample integrity. Serum albumin concentration was measured using the Siemens ADVIA Chemistry XPT automated biochemistry analyzer (Siemens Healthineers, Germany).

For serum preparation, blood samples were collected in red-cap tubes without anticoagulant and allowed to clot at room temperature for 30 minutes. Serum was separated by centrifugation at 3,000 rpm for 10 minutes and stored at -80°C until analysis.

#### Quality control

The laboratory adhered to the quality control system established by the National Center for Clinical Laboratories (NCCL). Daily instrument calibration was performed using manufacturer-provided controls, and internal quality control samples were analyzed to ensure measurement accuracy and precision. Unified reagent batches were used throughout the study period to minimize inter-assay variability. All analyses were performed in the same central laboratory using identical instruments and standardized protocols to ensure consistency across the entire cohort.

### Statistical analysis

Statistical analyses were conducted using R 4.3.3 and SPSS 25. Continuous variables with normal distribution were expressed as means and standard deviations, while categorical variables were expressed as percentages. Independent two-sample t-tests were used for continuous variables with normal distribution, and chi-square tests were used for categorical variables and quartile groups (Q1, Q2, Q3, and Q4) to compare baseline characteristics and incidence rates between breast cancer patients with and without distant metastasis. Three logistic regression models estimated the odds ratios (OR) and 95% confidence intervals (CI) for ALI as a continuous variable (ALI per IQR) or categorical variable (quartiles). These models explored the relationship between ALI and distant metastasis in breast cancer: the unadjusted model (Model 1); the model adjusted for age, education, insurance, hypertension, diabetes, marital status, residence, and menstrual status (Model 2); and the model further adjusted for age, education, insurance, hypertension, diabetes, marital status, residence, menstrual status, pathological type, histological grade, and molecular subtype (Model 3). Results were presented as odds ratios (OR) with 95% confidence intervals (CI). Additionally, restricted cubic spline functions were used to visualize the dose-response relationship between ALI and the risk of distant metastasis in breast cancer. Segmental logistic regression was used for threshold effect analysis. The receiver operating characteristic (ROC) curve was used to assess the predictive ability of ALI for distant metastasis risk, and the “additive” method determined the optimal cutoff point. Interaction analyses using the product term [ALI × (interaction term)] were performed to determine how different subgroups influenced the relationship between ALI and distant metastasis in breast cancer.

## Results

### Baseline characteristics

**Figure 1:**
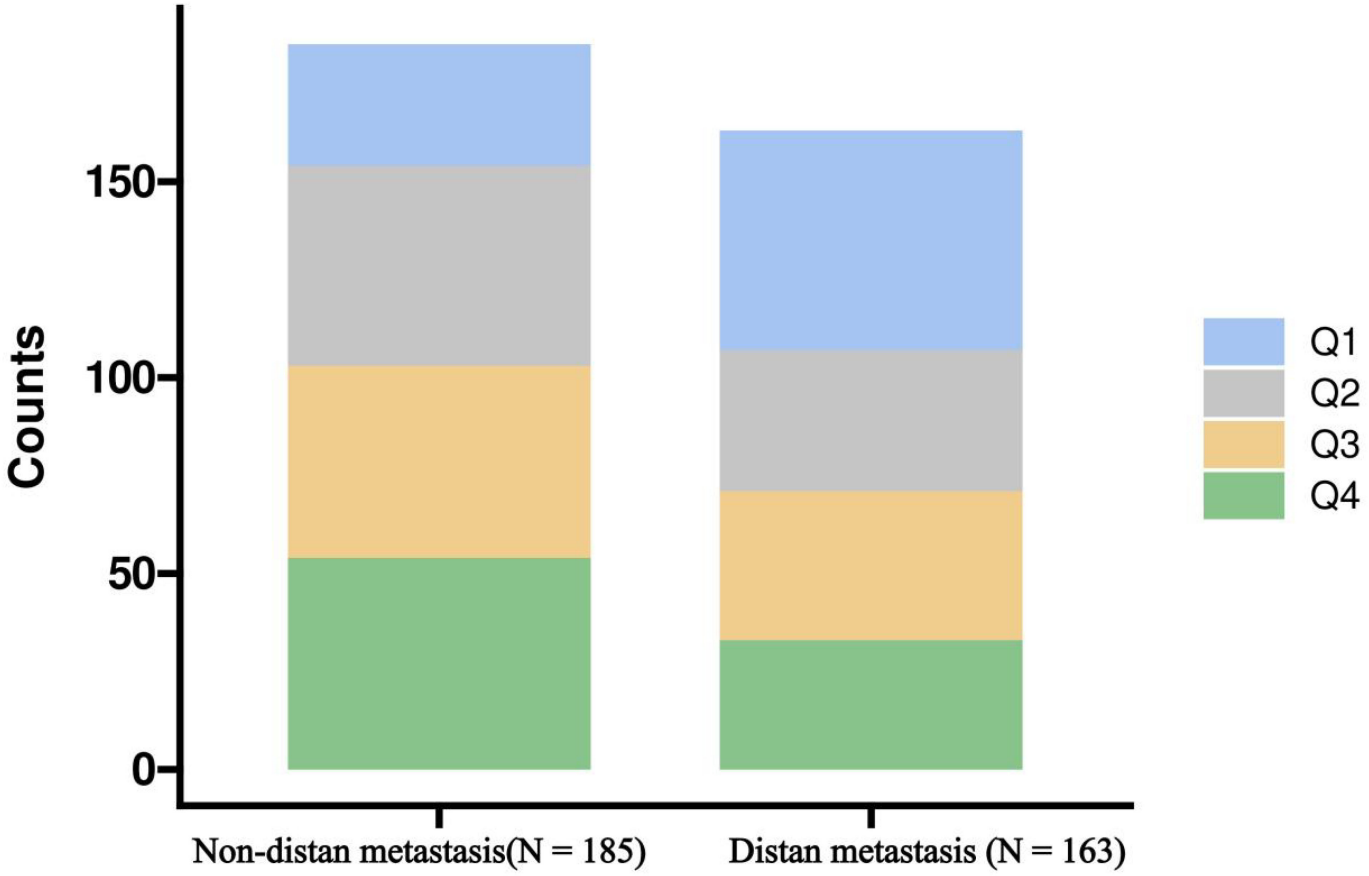
Distribution of Advanced Lung Cancer Inflammation Index (ALI) quartiles in breast cancer patients with and without distant metastasis, represented in a stacked bar chart. Q1, first quartiles of ALI; Q2, second quartiles of ALI; Q3, third quartiles of ALI; Q4 fourth quartiles of ALI.

**Table 1** provides important insights into the demographic and clinical characteristics of the study participants. The results revealed a significant age difference between non-metastatic breast cancer (47 ± 9 years) and metastatic breast cancer (51 ± 11 years) patients, with a p-value of 0.003. Significant differences were also observed between the two groups in education level, insurance status, residence, menstrual status, and breast cancer molecular subtype. Regarding comorbidities, the prevalence of hypertension was higher in patients with metastatic breast cancer (p=0.006). Additionally, significant differences were found between the two groups in BMI, neutrophil levels, serum albumin levels, neutrophil-to-lymphocyte ratio, and ALI (p<0.05). When ALI was categorized into quartiles (Q1-Q4), its distribution between the two groups was inversely related (**Figure 1**), with a p-value of 0.002. These findings suggest that ALI may play a role in distinguishing between the two groups.

### Dose–response relationship

**Table 2** shows the incremental ratio of ALI relative to IQR and the association between ALI quartiles and the risk of distant metastasis in breast cancer. Three models were constructed to examine the relationship between ALI and distant metastasis in breast cancer. When ALI per IQR was used as a continuous variable, Models 1, 2, and 3 indicated that the risk of distant metastasis decreased as ALI per IQR increased. When ALI quartiles were used as categorical variables, the risk of distant metastasis decreased with higher ALI quartiles (p for trend=0.003). After adjusting for age, education, insurance, hypertension, diabetes, marital status, residence, menstrual status, pathological type, histological grade, and molecular subtype, the highest ALI quartile (Q4) had the lowest risk of distant metastasis (OR, 0.28, 95% CI, 0.14-0.57, P<0.001) in Model 3.

**Table 2:** Association of ALI with the risk of Distant metastatic breast cancer.

Variable	Model 1 OR (95% CI)	P value	Model 2 OR (95% CI)	P value	Model 3 OR (95% CI)	P value
ALI per IQR	0.72 (0.56, 0.90)	0.007	0.71 (0.55, 0.91)	0.008	0.69 (0.52, 0.89)	0.005
Quartiles of ALI						
Q1	Ref		Ref		Ref	
Q2	0.39 (0.21,0.72)	0.003	0.38 (0.19, 0.72)	0.004	0.34 (0.17, 0.68)	0.003
Q3	0.43 (0.23, 0.79)	0.007	0.47 (0.24, 0.91)	0.025	0.42 (0.20, 0.83)	0.014
Q4	0.34 (0.18, 0.62)	<0.001	0.32 (0.16, 0.63)	<0.001	0.28 (0.14, 0.57)	<0.001
*p for trend*	0.002		0.004		0.003	

Model 1 was crude model. Model 2 was adjusted for age, education, insurance, hypertension, diabetes, marital status, location, and menstrual status. Model 3 was adjusted for age, education, insurance, hypertension, diabetes, marital status, location, menstrual status, pathological type, histological grade and subtype.

Key Finding: Higher ALI quartiles are associated with a progressively lower risk of distant metastasis, with the highest quartile (Q4) showing a 72% risk reduction compared to the lowest quartile (Q1) in the fully adjusted model. ALI, advanced lung cancer inflammation index; IQR, interquartile range.

**Figure 2:**
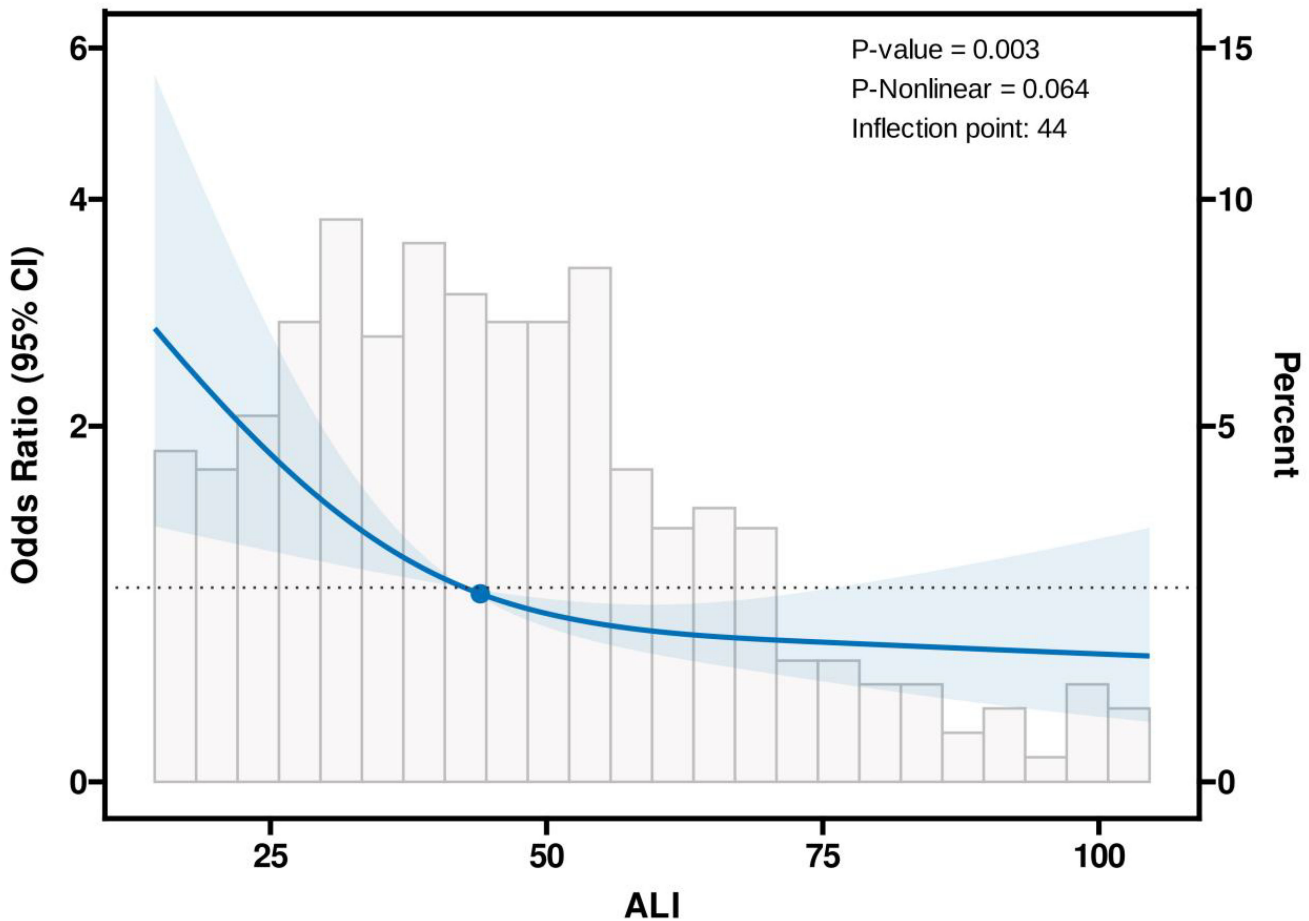
Association between the Advanced Lung Cancer Inflammation Index (ALI) and distant metastatic breast cancer using the Restricted Cubic Spline function. The model includes three knots positioned at the 10th, 50th, and 90th percentiles of ALI. The Y-axis represents the odds ratio (OR) for the presence of distant metastatic breast cancer at any given ALI value, relative to the reference value (50th percentile) of ALI. The logistic regression model was adjusted for age, education, insurance, hypertension, diabetes, marital status, geographic location, menstrual status, pathological type, histological grade, and subtype.

**Figure 2** illustrates the dose-response relationship between ALI and the risk of distant metastasis in breast cancer. Restricted cubic spline regression revealed a linear relationship between ALI and the risk of distant metastasis (non-linearity P=0.064). Segmental logistic regression further analyzed the threshold effect of ALI on distant metastasis (**Table 3**). When ALI < 44, the risk of distant metastasis decreased with increasing ALI (OR, 0.93, 95% CI, 0.90-0.96, P<0.001); however, when ALI ≥ 44, the association between ALI and distant metastasis was not statistically significant.

**Table 3:** Effect of ALI Level on Group: Adjusted Odds Ratios from Segmented Logistic Regression Analysis.

Characteristic	OR	95% CI	*P* value
ALI (< 44)	0.93	(0.90, 0.96)	<0.001
ALI (≥ 44)	0.99	(0.98, 1.01)	0.3

ORs were adjusted for age, education, insurance, hypertension, diabetes, marital status, location, menstrual status, pathological type, histological grade and subtype.

**Figure 3:**
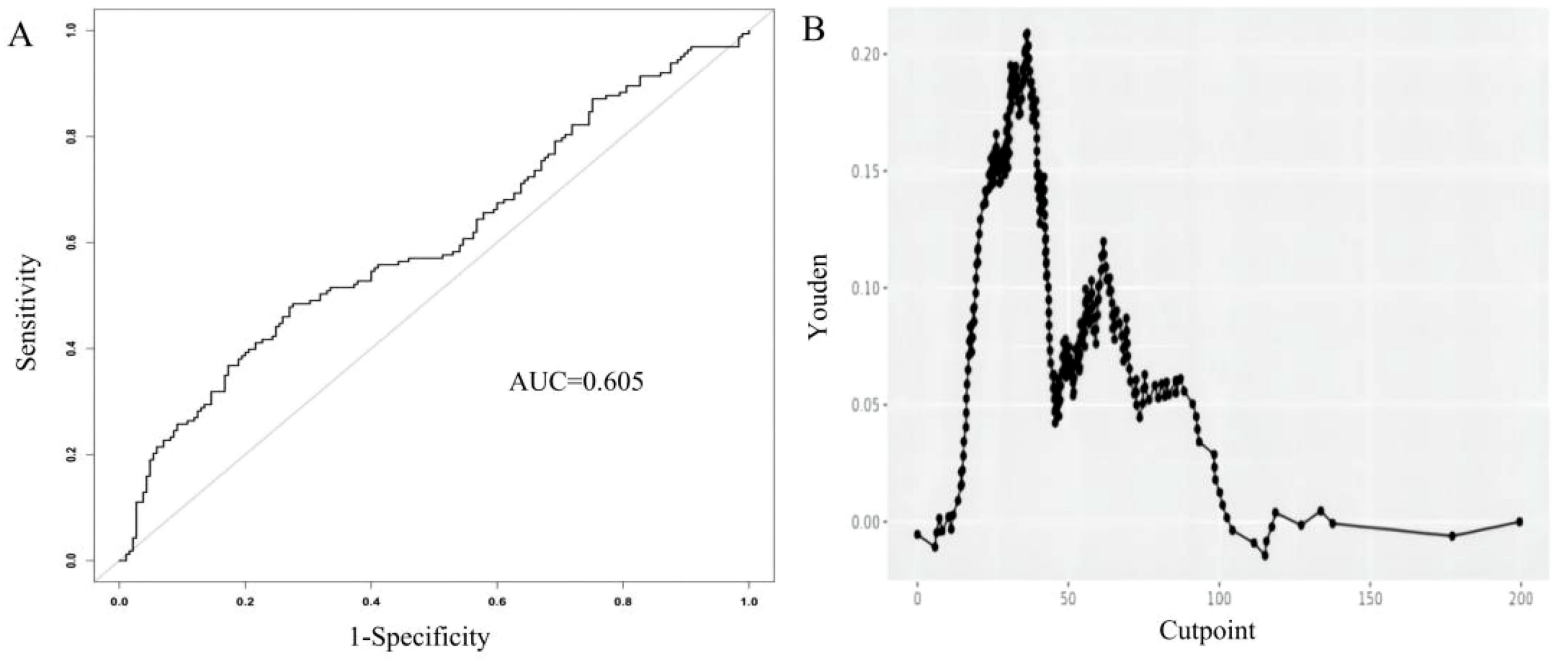
Receiver Operating Characteristic (ROC) curve for the Advanced Lung Cancer Inflammation Index (ALI) in predicting distant metastatic breast cancer **(A)**. The relationship between the cutpoint and the Youden index in the study. Optimal cutpoint (36.39) calculated based on the maximum Youden index (0.209) **(B)**.

When using ALI alone to predict distant metastasis in breast cancer, the AUC value of ALI was 0.605 (**Figure 3A**). The model showed optimal diagnostic performance when the Youden index was highest, with the best cutoff value at 36.39 (**Figure 3B**). Detailed parameters are provided in **Table 4**.

**Table 4:** Optimal cutpoint calculated based on the maximum Youden index.

AUC	Cutpoint	Metric score: youden	Sensitivity	Specificity	PPV	NPV	Accuracy
0.605	36.39	0.209	48.50%	72.40%	60.80%	61.50%	61.20%

AUC, Area Under the Curve; PPV, Positive Predictive Value; NPV, Negative Predictive Value.

### Stratified analysis

**Figure 4:**
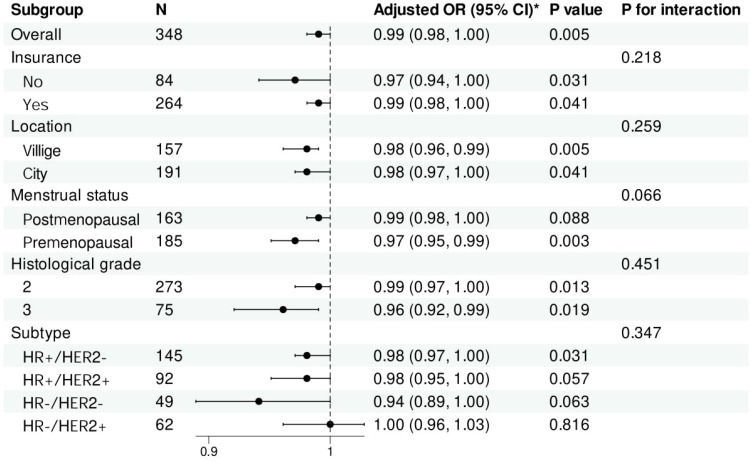
Forest plot of stratified analysis of the association of Advanced Lung Cancer Inflammation Index with the risk of distant metastatic breast cancer. *Adjusted for age, education, insurance, hypertension, diabetes, marital status, location, menstrual status, pathological type, histological grade and subtype. Histological grade: 2, Moderately differentiated; 3, Poorly differentiated. OR, odds ratio; CI, confidence intervals.

To understand whether the effect of ALI on the risk of distant metastasis in breast cancer varies across different subgroups, participants were stratified by characteristics. The results indicated that, after adjusting for all confounding factors, the impact of ALI on distant metastasis risk was consistent across different subgroups. No significant interactions were found between ALI and insurance status, residence, menstrual status, histological grade, or breast cancer molecular subtype (**Figure 4**).

## Discussion

Our baseline data reveal that patients with metastatic breast cancer tend to be older, less educated, less likely to have insurance, more frequently reside in rural areas, are more likely to be postmenopausal, and have a higher prevalence of hypertension and HR+/HER2- subtype. This study is the first to systematically investigate the relationship between ALI (Albumin-to-Lymphocyte Ratio) and metastatic breast cancer. Our findings indicate a significant association between lower ALI and the presence of distant metastases in breast cancer. After adjusting for confounding factors, each interquartile range (IQR) increase in ALI was associated with a 31% reduced risk of distant metastasis (OR, 0.69; 95% CI, 0.52-0.80). Dose-response analysis revealed a linear relationship between ALI and the risk of distant metastasis, with an optimal cutoff value of 36.39 and an AUC of 0.605. Stratified analysis showed that the relationship between ALI and distant metastasis was consistent across subgroups defined by insurance status, region, menopausal status, histological type, and molecular subtype.

When considering only the impact of Body Mass Index on distant metastasis, Mazzarella et al. found that obesity was significantly associated with poorer overall survival and increased incidence of distant metastases in ER-/HER2+ breast cancer (17). The promoting effect of obesity on metastasis is determined by tumor cells in the primary tumor rather than the microenvironment of secondary sites (18). However, in patients with diagnosed metastatic breast cancer, overweight and obesity are independent predictors of better overall survival in patients with good physical status. Higher BMI may help identify metastatic patients with superior survival outcomes (19, 20). The relationship between BMI and metastatic breast cancer at diagnosis is less clear. In our study, patients with distant metastases had lower BMI (p=0.018), which may be related to the higher energy demands of tumors with high malignancy potential. Additionally, more metastatic sites indicate a higher tumor burden. During cancer progression, tumors can reprogram host physiology, metabolism, and immune responses. Tumor-derived soluble factors, exosomes, and metabolites cause systemic changes in distant organs, facilitating metastasis and growth of cancer cells. These tumor-derived circulating factors can profoundly affect tissues with minimal metastatic cell habitation, such as skeletal muscle and adipose tissue. Indeed, many metastatic cancer patients develop a cachexia syndrome, characterized by muscle wasting and weakness (21). Studies have shown that lower serum albumin levels are associated with poorer survival rates in breast cancer and various cancers (22). Research on metastatic breast cancer has also identified low albumin levels as a predictor of metastasis at diagnosis (23). Our study found that metastatic breast cancer patients had lower serum albumin levels (p<0.001), consistent with previous findings. This may be attributed to decreased appetite, inadequate nutritional intake, and high tumor-related energy consumption in advanced cancer patients. Additionally, metastasis-induced systemic inflammation increases vascular permeability, leading to albumin leakage and breakdown. Elevated neutrophil counts in patients with metastatic breast cancer (p=0.046) are noteworthy. Studies suggest that high neutrophil counts may promote distant metastasis by enhancing angiogenesis, vascular remodeling, and tumor cell migration through protease-mediated extracellular matrix (ECM) and basement membrane degradation (24). Furthermore, high neutrophil counts and low serum albumin levels indicate poorer prognosis in metastatic breast cancer (25). Unlike previous research, our study integrates these variables to assess ALI, a variable not extensively studied in breast cancer. We demonstrated the significant predictive value of ALI for distant metastasis and confirmed its robustness across different cancer types.

In the past decade, metastatic breast cancer has imposed a significant economic burden, with 561,334 global deaths in 2015, most due to metastatic disease. By 2030, the mortality rate is expected to rise to 805,116 annually, representing a 43% increase in absolute deaths (26). Commercial surveys indicate that nearly two-thirds of breast cancer patients in China are diagnosed with advanced disease. In contrast, 60% of women in the US present with local stage I and II disease, 33% with regional stage III, and only 5% with stage IV disease. For newly diagnosed breast cancer, prolonged waiting times before initiating treatment impact prognosis, especially if delays result in stage progression and disease worsening or lead to more treatment complications (27). Currently, the identification and diagnosis of distant metastasis primarily rely on imaging techniques such as X-rays, bone scintigraphy, computed tomography (CT), magnetic resonance imaging (MRI), and positron emission tomography-computed tomography (PET-CT). X-ray is the most commonly used and cost-effective method but has lower sensitivity and may miss early metastases (28). Other imaging methods face issues such as uneven medical resource distribution (29), limited equipment availability, and high costs (30). Even in developed regions, excessive testing without prior evaluation can lead to longer hospital stays and increased costs (31). Thus, finding and studying new predictors for distant metastasis in breast cancer is crucial, especially in underdeveloped areas. Our study is the first to identify different quartile distributions of ALI between breast cancer patients with and without distant metastases. When ALI is treated as a categorical variable, after adjusting for confounding factors, we found that the fourth quartile group (Q4) had the lowest probability of distant metastasis (OR, 0.28; 95% CI, 0.14-0.27). Stratified analyses confirmed the consistent role of ALI in identifying distant metastasis across various populations.

The prognostic relationship between ALI and lung cancer has been established. Research on applying ALI to other cancers is gaining traction. In colorectal cancer, Kusunoki et al. identified the advanced lung cancer index as a useful prognostic indicator for patients undergoing colorectal cancer surgery (32). In gastric cancer, preoperative ALI was an independent prognostic factor for patients undergoing radical gastrectomy (33). Similarly, higher ALI has been significantly associated with better prognosis in patients with advanced pancreatic cancer undergoing concurrent chemoradiotherapy (34). ALI also provides significant predictive value for patients with metastatic melanoma receiving immunotherapy as second-line treatment (35). Tsai found ALI to be a promising prognostic biomarker for patients with oral squamous cell carcinoma undergoing initial surgery; additionally, ALI-based nomograms may be useful for individualized OS and DFS estimates (36). Despite limited studies on ALI and breast cancer, we are the first to explore its dose-response relationship with the risk of distant metastasis in breast cancer as a continuous variable. We developed three models to adjust for 11 factors (age, education, insurance, hypertension, diabetes, marital status, location, menstrual status, pathological type, histological grade, and subtype), ensuring the reliability and validity of our findings. We conducted stratified analyses to explore differences in the association between ALI and distant metastasis across multiple subgroups.

Our study has limitations. First, although we adjusted for multiple covariates, other potential confounders such as socioeconomic status and dietary habits may still affect the results. Second, the single-center design with a relatively small sample size necessitates further research to generalize our findings to the broader population. Lastly, due to insufficient follow-up time, we were unable to include prognostic data in this study. Future research should further investigate the impact of ALI on prognosis.

## Conclusion

Our study found a linear negative correlation between ALI and distant metastasis in breast cancer patients after adjusting for potential confounding factors. Future research, including randomized controlled trials or cohort studies, is urgently needed to validate these findings and provide more robust evidence for disease diagnosis.

## Data Availability

The original contributions presented in the study are included in the article/supplementary material. Further inquiries can be directed to the corresponding author.

## References

[1] Bray F Laversanne M Sung H Ferlay J Siegel RL Soerjomataram I. Global cancer statistics 2022: GLOBOCAN estimates of incidence and mortality worldwide for 36 cancers in 185 countries. CA A Cancer J Clin. (2024) 74:229–63. doi: 10.3322/caac.21834, PMID: 38572751

[2] Cao W Chen HD Yu YW Li N Chen WQ. Changing profiles of cancer burden worldwide and in China: a secondary analysis of the global cancer statistics 2020. Chin Med J (Engl). (2021) 134:783–91. doi: 10.1097/CM9.0000000000001474, PMID: 33734139 PMC8104205

[3] Siegel R Miller KD Wagle NS Jemal A. Cancer statistics, 2023. CA A Cancer J Clin. (2023) 73:17–48. doi: 10.3322/caac.21763, PMID: 36633525

[4] Joko-Fru WY Griesel M Mezger NCS Hämmerl L Seraphin TP Feuchtner J. Breast cancer diagnostics, therapy, and outcomes in Sub-Saharan Africa: A population-based registry study. J Natl Compr Canc Netw. (2021) 19:1–11. doi: 10.6004/jnccn.2021.7011, PMID: 34965508

[5] Gogate A Wheeler SB Reeder-Hayes KE Ekwueme DU Fairley TL Drier S. Projecting the prevalence and costs of metastatic breast cancer from 2015 through 2030. JNCI Cancer Spectr. (2021) 5:pkab063. doi: 10.1093/jncics/pkab063, PMID: 34409255 PMC8364673

[6] Lim B Hortobagyi G. Current challenges of metastatic breast cancer. Cancer Metastasis Rev. (2016) 35:495–514. doi: 10.1007/s10555-016-9636-y, PMID: 27933405

[7] Pesapane F Downey K Rotili A Cassano E Koh D-M. Imaging diagnosis of metastatic breast cancer. Insights Imaging. (2020) 11:79. doi: 10.1186/s13244-020-00885-4, PMID: 32548731 PMC7297923

[8] Zhan CH Liu GJ. Diagnostic value of a combined serum α-hydroxybutyrate dehydrogenase, carcinoembryonic antigen and glycoantigen 125 test for early-stage breast cancer. Breast Cancer (Dove Med Press). (2023) 15:617–23. doi: 10.2147/BCTT.S410500, PMID: 37600671 PMC10439733

[9] Zhang J Wei Q Dong D Ren L. The role of TPS, CA125, CA15–3 and CEA in prediction of distant metastasis of breast cancer. Clinica chimica Acta. (2021) 523:19–25. doi: 10.1016/j.cca.2021.08.027, PMID: 34454906

[10] Koizumi M Takahashi S Ogata E. Bone metabolic markers in bone metastasis of breast cancer. Int J Clin Oncol. (1999) 4:331–7. doi: 10.1007/s101470050080

[11] Li Y Chen Y Shao B Liu J Hu R Zhao F. Evaluation of creatine kinase (CK)-MB to total CK ratio as a diagnostic biomarker for primary tumors and metastasis screening. Pract Lab Med. (2023) 37:e00336. doi: 10.1016/j.plabm.2023.e00336, PMID: 37767053 PMC10520525

[12] Song M Zhang Q Song C Liu T Zhang X Ruan G. The advanced lung cancer inflammation index is the optimal inflammatory biomarker of overall survival in patients with lung cancer. J cachexia sarcopenia muscle. (2022) 13:2504–14. doi: 10.1002/jcsm.13032, PMID: 35833264 PMC9530543

[13] Jafri S Shi R Mills G. Advance lung cancer inflammation index (ALI) at diagnosis is a prognostic marker in patients with metastatic non-small cell lung cancer (NSCLC): a retrospective review. BMC Cancer. (2013) 13:158. doi: 10.1186/1471-2407-13-158, PMID: 23530866 PMC3618002

[14] He X Zhou T Yang Y Hong S Zhan J Hu Z. Advanced lung cancer inflammation index, a new prognostic score, predicts outcome in patients with small-cell lung cancer. Clin Lung Cancer. (2015) 16:e165–71. doi: 10.1016/j.cllc.2015.03.005, PMID: 25922292

[15] Hua X Chen J Wu Y Sha J Han S Zhu X-L. Prognostic role of the advanced lung cancer inflammation index in cancer patients: a meta-analysis. World J Surg Onc. (2019) 17:177. doi: 10.1186/s12957-019-1725-2, PMID: 31677642 PMC6825711

[16] Mountzios G Samantas E Senghas K Zervas E Krisam J Samitas K. Association of the advanced lung cancer inflammation index (ALI) with immune checkpoint inhibitor efficacy in patients with advanced non-small-cell lung cancer. ESMO Open. (2021) 6:100254. doi: 10.1016/j.esmoop.2021.100254, PMID: 34481329 PMC8417333

[17] Mazzarella L Disalvatore D Bagnardi V Rotmensz N Galbiati D Caputo S. Obesity increases the incidence of distant metastases in oestrogen receptor-negative human epidermal growth factor receptor 2-positive breast cancer patients. Eur J Cancer. (2013) 49:3588–97. doi: 10.1016/j.ejca.2013.07.016, PMID: 23953055

[18] Bousquenaud M Fico F Solinas G Rüegg C Santamaria-Martínez A. Obesity promotes the expansion of metastasis-initiating cells in breast cancer. Breast Cancer Res. (2018) 20:104. doi: 10.1186/s13058-018-1029-4, PMID: 30180888 PMC6123990

[19] Tsang NM Pai PC Chuang CC Chuang WC Tseng CK Chang KP. Overweight and obesity predict better overall survival rates in cancer patients with distant metastases. Cancer Med. (2016) 5:665–75. doi: 10.1002/cam4.634, PMID: 26811258 PMC4831285

[20] Saleh K Carton M Dieras V Heudel P-E Brain E D’hondt V. Impact of body mass index on overall survival in patients with metastatic breast cancer. Breast. (2020) 55:16–24. doi: 10.1016/j.breast.2020.11.014, PMID: 33307392 PMC7725947

[21] Biswas AK Acharyya S. Understanding cachexia in the context of metastatic progression. Nat Rev Cancer. (2020) 20:274–84. doi: 10.1038/s41568-020-0251-4, PMID: 32235902

[22] Gupta D Lis C. Pretreatment serum albumin as a predictor of cancer survival: A systematic review of the epidemiological literature. Nutr J. (2010) 9:69–9. doi: 10.1186/1475-2891-9-69, PMID: 21176210 PMC3019132

[23] Yur M. Comparison of metastatic versus non-metastatic breast cancer at the time of diagnosis and risk factors for primary metastatic breast cancer. Ann Med Res. (2023) 30:1. doi: 10.5455/annalsmedres.2023.08.184

[24] Mouchemore KA Anderson RL Hamilton J. Neutrophils, G-CSF and their contribution to breast cancer metastasis. FEBS J. (2018) 285:665–79. doi: 10.1111/febs.14206, PMID: 28834401

[25] Xiang M Zhang H Tian J Yuan Y Xu Z Chen J. Low serum albumin levels and high neutrophil counts are predictive of a poorer prognosis in patients with metastatic breast cancer. Oncol Lett. (2022) 24(6):432. doi: 10.3892/ol.2022.13552, PMID: 36311691 PMC9608081

[26] Cardoso F Spence D Mertz S Corneliussen-James D Sabelko K Gralow J. Global analysis of advanced/metastatic breast cancer: Decade report (2005-2015). Breast. (2018) 39:131–8. doi: 10.1016/j.breast.2018.03.002, PMID: 29679849

[27] Fan L Strasser-Weippl K Li JJ St Louis J Finkelstein DM Yu K-D. Breast cancer in China. Lancet Oncol. (2014) 15:e279–289. doi: 10.1016/S1470-2045(13)70567-9, PMID: 24872111

[28] Hamaoka T Madewell JE Podoloff DA Hortobagyi GN Ueno NT. Bone imaging in metastatic breast cancer. JCO. (2004) 22:2942–53. doi: 10.1200/JCO.2004.08.181, PMID: 15254062

[29] Ding L Zhang N Mao Y. Addressing the maldistribution of health resources in Sichuan Province, China: A county-level analysis. PLoS One. (2021) 16:e0250526. doi: 10.1371/journal.pone.0250526, PMID: 33891649 PMC8064550

[30] Saini S Sharma R Levine L Barmson RT Jordan P Thrall J. Technical cost of CT examinations. Radiology. (2001) 218:172–5. doi: 10.1148/radiology.218.1.r01ja01172, PMID: 11152797

[31] Hendee W Becker G Borgstede J Bosma J Casarella WJ Erickson BA. Addressing overutilization in medical imaging. Radiology. (2010) 257:240–5. doi: 10.1148/radiol.10100063, PMID: 20736333

[32] Kusunoki K Toiyama Y Okugawa Y Yamamoto A Omura Y Ohi M. Advanced lung cancer inflammation index predicts outcomes of patients with colorectal cancer after surgical resection. Dis Colon Rectum. (2020) 63:1242–50. doi: 10.1097/DCR.0000000000001658, PMID: 33216495

[33] Zhang X Wang D Sun T Li W Dang C. Advanced lung cancer inflammation index (ALI) predicts prognosis of patients with gastric cancer after surgical resection. BMC Cancer. (2022) 22(1):684. doi: 10.1186/s12885-021-09064-0, PMID: 35729545 PMC9215041

[34] Topkan E Mertsoylu H Ozdemir Y Sezer A Kucuk A Besen AA. Prognostic usefulness of advanced lung cancer inflammation index in locally-advanced pancreatic carcinoma patients treated with radical chemoradiotherapy. CMAR. (2019) 11:8807–15. doi: 10.2147/CMAR.S222297, PMID: 31632140 PMC6789411

[35] Cheng X Dong Y Lou F. The predictive significance of the advanced lung cancer inflammation index (ALI) in patients with melanoma treated with immunotherapy as second-line therapy. CMAR. (2021) 13:173–80. doi: 10.2147/CMAR.S286453, PMID: 33469361 PMC7810587

[36] Tsai Y Hsu C-M Chang G-H Tsai M-S Lee Y-C Huang E-I. Advanced lung cancer inflammation index predicts survival outcomes of patients with oral cavity cancer following curative surgery. Front Oncol. (2021) 11:609314. doi: 10.3389/fonc.2021.609314, PMID: 34660250 PMC8514840

